# Spectrophotometric Determination of Molybdenum(VI) as a Ternary Complex with 4-Nitrocatechol and Benzalkonium Chloride

**DOI:** 10.3390/molecules27041217

**Published:** 2022-02-11

**Authors:** Vidka V. Divarova, Antoaneta Saravanska, Galya Toncheva, Nikolina Milcheva, Vassil B. Delchev, Kiril Gavazov

**Affiliations:** 1 Department of Chemical Sciences, Faculty of Pharmacy, Medical University of Plovdiv, 120 Buxton Bros Str., 4004 Plovdiv, Bulgaria; vidka.divarova@mu-plovdiv.bg (V.V.D.); antoaneta.saravanska@mu-plovdiv.bg (A.S.); nikolina.milcheva@mu-plovdiv.bg (N.M.); 2 Faculty of Chemistry, University of Plovdiv ‘Paisii Hilendarskii’, 24 Tsar Assen St., 4004 Plovdiv, Bulgaria; galq_toncheva@mail.bg (G.T.); vdelchev@uni-plovdiv.net (V.B.D.)

**Keywords:** molybdenum, liquid—liquid extraction, 4-nitrocatechol, benzalkonium chloride, spectrophotometric determination, TD DFT calculations

## Abstract

A new liquid—liquid extraction system for molybdenum(VI) was studied. It contains 4-nitrocatechol (4NC) as a complexing chromogenic reagent and benzalkonium chloride (BZC) as a source of heavy cations (BZ^+^), which are prone to form chloroform-extractable ion-association complexes. The optimum conditions for the determination of trace molybdenum(VI) were found: concentrations of 4NC and BZC (7.5 × 10^−4^ mol dm^−3^ and 1.9 × 10^−4^ mol dm^−3^, respectively), acidity (3.75 × 10^−2^ mol dm^−3^ H_2_SO_4_), extraction time (3 min), and wavelength (439 nm). The molar absorptivity, limit of detection, and linear working range were 5.5 × 10^4^ dm^3^ mol^−1^ cm^−1^, 5.6 ng cm^−3^, and 18.6–3100 μg cm^−3^, respectively. The effect of foreign ions was examined, and the developed procedure was applied to the analysis of synthetic mixtures and real samples (potable waters and steels). The composition of the extracted complex was 1:1:2 (Mo:4NC:BZ). Three possible structures of its anionic part [Mo^VI^(4NC)O_2_(OH)_2_]^2−^ were discussed based on optimizations at the B3LYP/3-21G level of theory, and simulated UV/Vis absorption spectra were obtained with the TD Hamiltonian.

## 1. Introduction

Molybdenum is a second-row transition metal that belongs to group six and occupies position 42 in the periodic table. It is a silvery-white refractory metal with a high thermal and electrical conductivity, low vapor pressure, low coefficient of thermal expansion, and good alloyability with ferrous and nonferrous metals. Its compounds are important for a number of industries, but the majority of molybdenum produced (over 250,000 tons in 2018 [1] is used in steels and alloys [2,3]. Molybdenum’s role in such materials is to improve the hardness, strength, ductility, and resistance to shock, fatigue, and creep, especially at elevated temperatures.

Molybdenum is a relatively rare element in the continental Earth’s crust (average content of 1.2 mg kg^−1^) [4] and fresh waters [5,6]. However, it is essential for microorganisms, plants, and animals [7]. More than 50 molybdenum-dependent enzymes are known in all kingdoms of life. Important for humans are four enzymes with a pterin-based cofactor [8]. Their synthesis and function depend on many factors, including diet [5]. According to some metabolic balance studies, the adequate molybdenum intake for healthy people (over the age of 15) is 65 μg per day [9].

Various techniques have been used for the determination of molybdenum in environmental and industrial samples, e.g., inductively coupled plasma mass spectrometry, inductively coupled plasma optical emission spectrometry, electrothermal atomic absorption spectrometry, and spectrophotometry [10,11,12,13]. Spectrophotometry is a simple, cheap, convenient, and mature analytical technique [14,15,16]. It can be easily combined with extraction methods [17,18,19,20,21,22,23] to improve analytical performance.

The aim of the present work is to develop a sensitive and selective extractive spectrophotometric procedure for the determination of molybdenum in steels and potable waters with 4-nitrocatechol (4NC) and benzalkonium chloride (BZC). An effective ligand for the formation of colored complexes that are attractive for analytical applications is 4-nitrocatechol [24,25,26,27]. Benzalkonium chloride is a mixture of alkyl dimethyl benzyl ammonium chlorides [28,29], with an average molar mass of 360 g mol^−1^. Recently, it has been used in our laboratory as a liquid—liquid extraction reagent for cobalt [30].

## 2. Results and Discussion

### 2.1. Liquid—Liquid Extraction—Spectrophotometric Optimization

**Figure 1:**
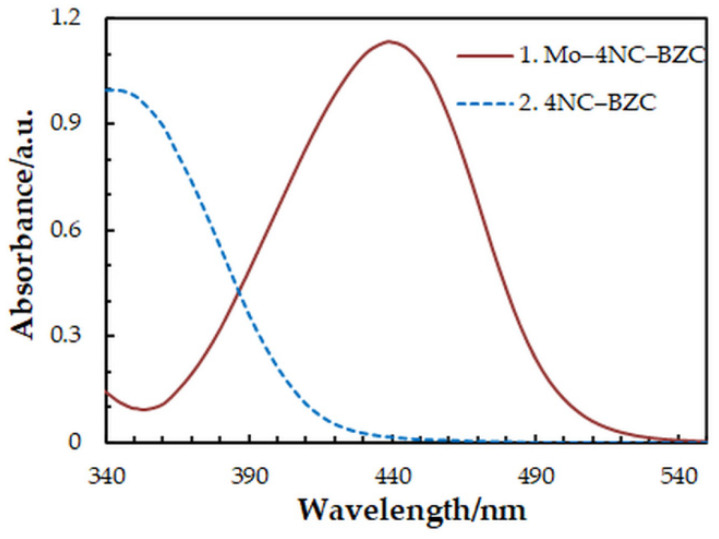
Absorption spectra of the complex (1) and blank (2): *c*_Mo_ = 2 × 10^−5^ mol dm^−3^, *c*_4NC_ = 7.5 × 10^−4^ mol dm^−3^, *c*_BZC_ = 1.9 × 10^−4^ mol dm^−3^, *c*_H2SO4_ = 3.75 × 10^−2^ mol dm^−3^, *t*_ex_ = 3 min.

**Figure 2:**
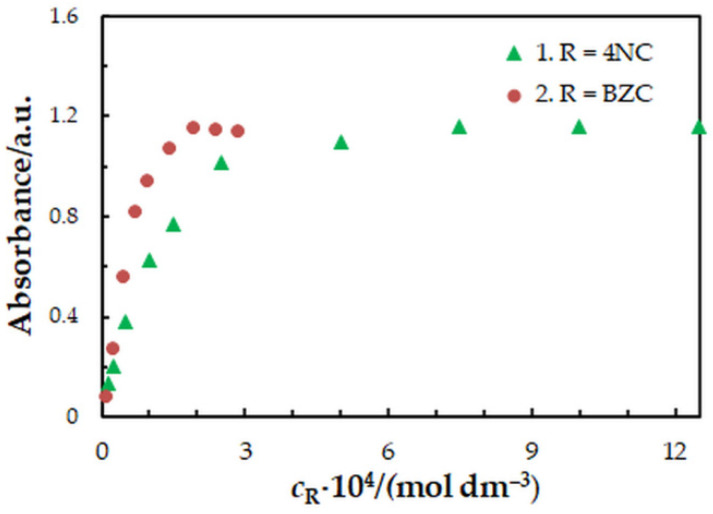
Effect of the 4NC (1) and BZC (2) concentration: *c*_Mo_ = 2 × 10^−5^ mol dm^−3^, *t*_ex_ = 3 min, *λ* = 439 nm. 1—*c*_BZC_ = 1.9 × 10^−4^ mol dm^−3^; 2—*c*_4NC_ = 7.5 × 10^−4^ mol dm^−3^.

**Figure 3:**
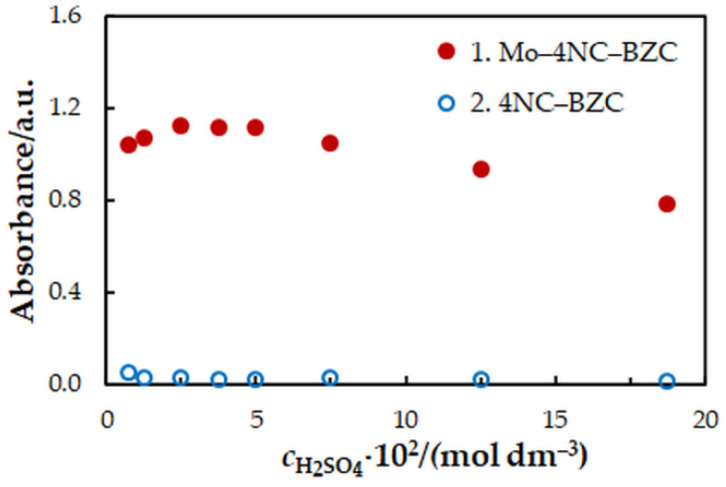
Effect of the H_2_SO_4_ concentration: *c*_Mo_ = 2 × 10^−5^ mol dm^−3^, *c*_4NC_ = 7.5 × 10^−4^ mol dm^−3^, *c*_BZC_ = 1.9 × 10^−4^ mol dm^−3^, *t*_ex_ = 3 min, *λ* = 439 nm.

**Figure 4:**
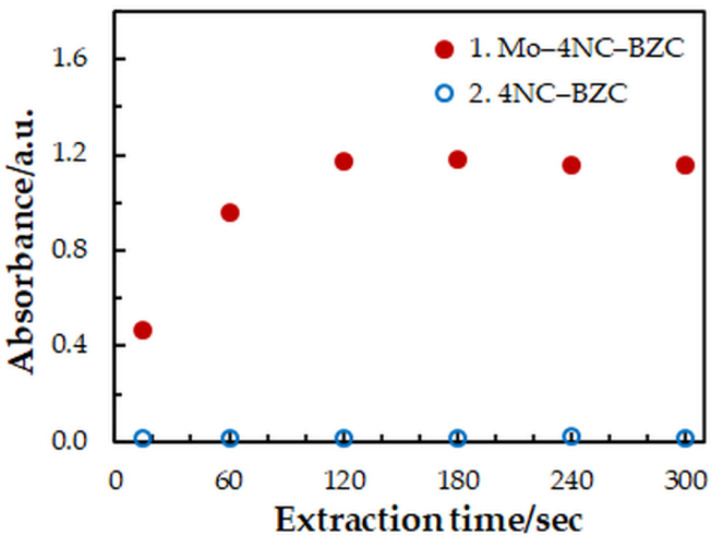
Effect of the extraction time: *c*_Mo_ = 2 × 10^−5^ mol dm^−3^, *c*_4NC_ = 7.5 × 10^−4^ mol dm^−3^, *c*_BZC_ = 1.9 × 10^−4^ mol dm^−3^, *c*_H2SO4_ = 3.75 × 10^−2^ mol dm^−3^, *λ* = 439 nm.

One-factor-at-a-time optimization was carried out at room temperature to find the optimum values of the following extraction—spectrophotometric parameters: wavelength of spectrophotometric measurement (Figure 1), concentration of 4NC (Figure 2), concentration of BZC (Figure 2), concentration of H_2_SO_4_ (Figure 3), and extraction time (Figure 4). Under the optimum conditions (Table 1), the complex has an absorption maximum at 439 nm, where the blank absorbs insignificantly.

### 2.2. Molar Ratios and Formula of the Ternary Complex

The complexation in acidic medium between the tetrahedral molybdate MoO_4_^2−^ and reagents bearing a catechol moiety (L) leads to the production of octahedral anionic products in which the molar L-to-Mo ratio is 1-to-1: [Mo^VI^O_3_(OH)L]^3−^, [Mo^VI^O_2_(OH)_2_L]^2−^, and [Mo^VI^O(OH)_3_L]^−^. As the acidity decreases, complexes with a molar ratio of 2:1 (L:Mo) are also formed [31,32,33].

**Figure 5:**
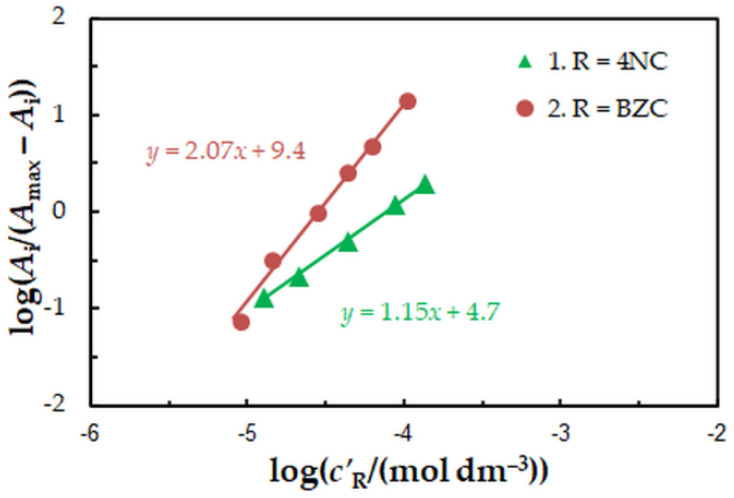
Determination of the 4NC:Mo (1) and BZC:Mo (2) molar ratios by the mobile equilibrium method. The data are derived from the experimental points in Figure 2.

**Figure 6:**
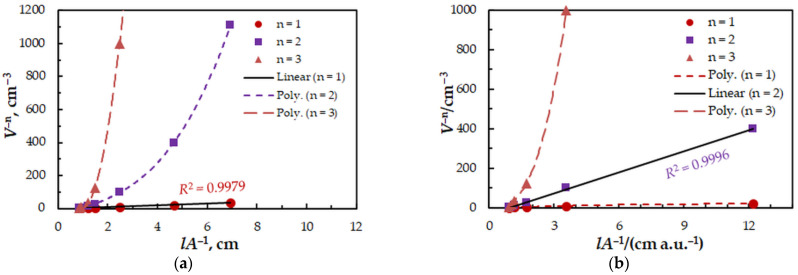
Determination of the 4NC:Mo (**a**) and BZC:Mo (**b**) molar ratios by the straight-line method of Asmus. The data are derived from the experimental points in Figure 2.

To find the 4NC-to-Mo(VI) and BZC-to-Mo(VI) molar ratios in the examined ternary complex, we used the mobile equilibrium method [34], and the straight-line method of Asmus [35]. The results (Figure 5 and Figure 6) show a composition of 1:1:2 (Mo:4NC:BZC). Since BZC forms monovalent cations (BZ^+^), the ternary complex can be represented by the following formula: (BZ^+^)_2_[Mo^VI^O_2_(OH)_2_(4NC)].

### 2.3. Extraction Constant, Distribution Ratio and Fraction Extracted

The equation of ion-association and subsequent extraction of the ternary complex is as follows:

[Mo^VI^(4NC)O_2_(OH)_2_]^2−^_(aq)_ + 2BZ^+^_(aq)_ ↔ (BZ^+^)_2_[Mo^VI^(4NC)O_2_(OH)_2_]_(org)_

The equilibrium constant characterizing this equation was determined by three methods based on the saturation curve with BZC (Figure 2, series 2): the mobile equilibrium method [34] (Figure 5, straight line 2), the Holme—Langmyhr method [36], and the Harvey—Manning method [37]. The obtained values are given in Table 2, along with values of other extraction characteristics: distribution ratio (*D*) and fraction extracted (*E*). The fact that the extraction constants (*K*) obtained by the above-mentioned methods (which operate with points located in different sections of the experimental saturation curve) are statistically identical, shows that the proposed equation is correct and there are no significant side processes.

### 2.4. Ground-State Equilibrium Geometries of the Anionic Part, Spectral Comparison, Energies, and Kinetics

**Figure 7:**
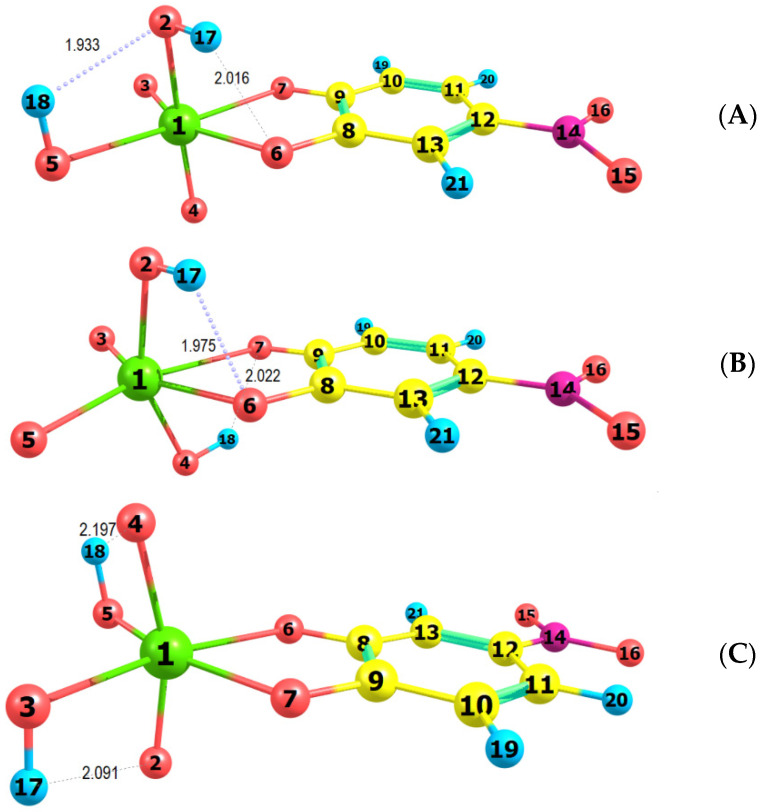
Optimized ground-state equilibrium geometries of the three possible structures, (**A**–**C**).

**Figure 8:**
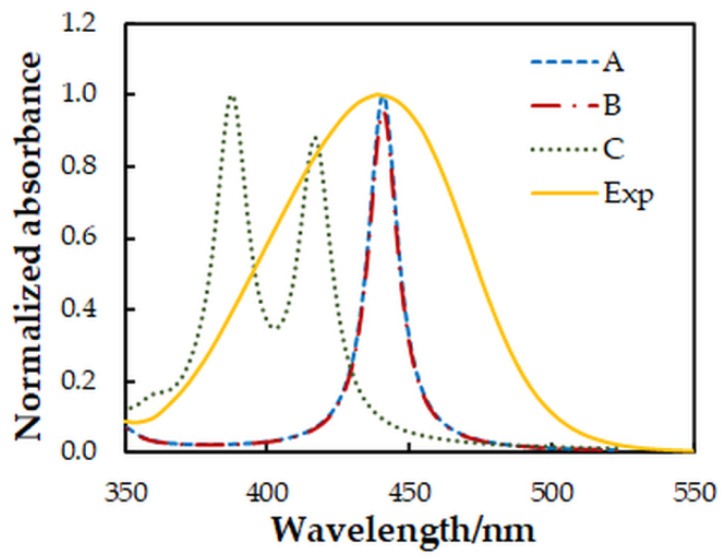
Comparison between experimental (Exp) and simulated (A–C) absorption spectra. A Lorentzian broadening and a scaling factor of 0.76 were used for the theoretical spectra.

The spectral characteristics of the ion-association complex in the visible range are determined mainly by its anionic part [27]. Therefore, it is interesting to make a comparison between the experimental spectrum and simulated spectra of different isomers of the complex anion [Mo^VI^O_2_(OH)_2_(4NC)]^2−^. For this purpose, the ground-state equilibrium geometries of three possible structures of the anionic part were optimized at the B3LYP/3-21G level of theory (Figure 7). Then, the vertical excitation energies were calculated with the TD Hamiltonian to simulate theoretical UV/Vis absorption spectra (Figure 8).

Figure 7 shows that two intramolecular H-bonds are observed in each of the examined structures. The shortest are the H_18_…O_2_ (1.933 Å) in structure **A** and H_17_…O_6_ (1.975 Å) in structure **B**. The two hydrogen atoms in structure **B** are located at oxygens outside the 4NC plane (axial position) and form H-bonds with the catechol oxygens. The octahedron in this structure is the most distorted: <C_9_O_7_Mo_1_O_3_ = 168.9°, <O_2_Mo_1_O_4_ = 153.9°. The least distorted is the octahedron in structure **C (**<C_9_O_7_Mo_1_O_3_ = 179.9°, <O_2_Mo_1_O_4_ = 163.5°).

The comparison of the spectra (Figure 8) suggests that structures **A** and **B** are more probable than structure **C**. Further energy analysis shows that **B** is more stable than **A**. The energy difference between the two structures is 21 kJ mol^−1^. The changes in the standard enthalpy ΔH° and Gibbs free energy ΔG° for the transition of H_18_ from O_5_ to O_4_ are −21.82 kJ mol^−1^ and −21.88 kJ mol^−1^, respectively. The rate constants, calculated using the Eyring equation (transmission coefficient equal to unity) after the optimization of the transition state of the reaction, are *k*_forward_ = 8.45 × 10^−8^ s^−1^ and *k*_reverse_ = 1.24 × 10^−11^ s^−1^. This also gives grounds to conclude that the anionic part of the ternary complex is dominated by structure **B**.

### 2.5. Effect of Foreign Ions and Masking Agents

The effect of foreign ions was studied under the optimum conditions. The results are shown in Table 3. The most serious interferences are caused by W(VI), which forms stable chloroform-extractable complexes with 4NC and cationic ion-association reagents [38]. Therefore, in the analysis of tungsten-containing samples (e.g., steels), the sample preparation methodology must include a step for its separation (as described below).

It was found that Na_2_EDTA is an effective masking agent for several ions, including Fe(III). This makes it possible to apply the proposed procedure for the determination of molybdenum in steels and environmental samples.

### 2.6. Analytical Characteristics and Application

The relationship between the absorbance and the Mo(VI) concentration was studied under the optimal conditions. A good linearity was observed up to 3100 ng cm^−3^ Mo(VI) (*R*^2^ = 0.9995, *n* = 11). The linear regression equation was *A* = 0.577*γ* + 0.007, where *γ* is the mass concentration (μg cm^−3^). The standard deviations of the slope and intercept were 0.004 and 0.008, respectively. The molar absorption coefficient was 5.5 × 10^4^ dm^3^ mol^−1^ cm^−1^. The limit of detection (LOD) and limit of quantitation (LOQ) calculated as 3- and 10-times standard deviation of the blank divided by the slope, were 5.6 ng cm^−3^ and 18.6 ng cm^−3^, respectively. 

The developed procedure was applied to the analysis of referent standard steels (RSS) and synthetic mixtures (SM), imitating typical molybdenum-containing steels [2]. The results are given in Table 4. The relative standard deviation (RSD) for these determinations was in the range of 0.8–2.4%.

Commercial potable water from three Bulgarian brands was also a subject of analysis. The result obtained for the “Devin” mineral water (20 ng cm^−3^, *n* = 4, RSD = 10%) was confirmed by the standard addition method (22.5 ng cm^−3^, RSD = 5.3%) at three spiked concentration levels. The Mo(VI) content in the “Sevtopolis” table water and “Gorna Banya” mineral water was below the limit of determination.

### 2.7. Comparison with Other Liquid—Liquid Extraction–Spectrophotometric Procedures

A comparison of the present method with other liquid—liquid extraction–spectrophotometric methods for molybdenum determination is made in Table 5. The proposed method is characterized by a low LOD, high molar absorption coefficient, and good linearity. It is reliable and robust because of the wide optimum intervals of the variables studied. In addition, the volume of organic solvent used (5 cm^3^ per sample) is smaller than that of most of the procedures described in Table 5.

## 3. Experimental Section

### 3.1. Reagents and Chemicals

The solution of Mo(VI) (2 × 10^−4^ mol dm^−3^) was prepared from (NH_4_)_6_Mo_7_O_24_⋅4H_2_O (99.98% trace metals basis, Merck, Schnelldorf, Germany). The other chemicals were 4NC (> 98%, Fluka AG, Buchs, Switzerland), BZC (>95.0%, Merck, Schnelldorf, Germany), sulfuric acid (puriss. p. a., Fluka AG, Germany), and disodium ethylenediaminetetraacetate dihydrate (Na_2_EDTA; > 99.5%, Fillab EOOD, Plovdiv, Bulgaria) The prepared aqueous solutions were at concentrations of 7.5 × 10^−3^ mol dm^−3^ (4NC), 2.4 × 10^−3^ mol dm^−3^ (BZC), 2.5 × 10^−1^ mol dm^−3^ (H_2_SO_4_), and 1.0 × 10^−1^ mol dm^−3^ (Na_2_EDTA). Distilled or deionized (18.2 MΩ cm, ELGA-Veolia LabWater, High Wycombe, UK) water and redistilled chloroform were used throughout the work.

### 3.2. Instrumentation

Absorbance was measured on an Ultrospec 3300 pro UV/Vis spectrophotometer (Little Chalfont, UK), equipped with 10 mm path-length quartz cells. The pH was checked using a WTW InoLab 720 pH-meter (Weilheim, Germany).

### 3.3. General Optimization Procedure

Solutions of Mo(VI), H_2_SO_4_, 4NC, and BZC were successively transferred into a separatory funnel. The total volume was adjusted to 10 cm^3^ with water. Then, 5 cm^3^ of chloroform was added and the mixture was shaken for a fixed period (15–300 s). After a short wait for phase separation, a portion of the organic layer was poured into the cell. The absorbance was measured against chloroform or a blank prepared at the same manner.

### 3.4. Determination of the Distribution Ratio and Fraction Extracted

The distribution ratio (*D*) and fraction extracted (%*E*) were calculated by comparing the absorbances obtained after a single extraction (*A*_1_) and a triple extraction (*A*_3_) under the optimal conditions given in Table 1. The total volume in both cases (single extraction and triple extraction) was 25 cm^3^. The following formulae were used:

*D* = *A*_1_/(*A*_3_ − *A*_1_) (1)

%*E* = 100 × *D*/(*D* + 1). (2)

### 3.5. Sample Preparation

The steels were prepared for analysis according to a procedure described previously [47,48]. A known amount of the sample (250–1000 μg) was dissolved in 100 cm^3^ of HCl (1:1). Then, 20 cm^3^ of HNO_3_ (1:1) was added and the mixture was heated for 5 min or until tungstic acid became light yellow (incase the steel contains tungsten). The mixture was diluted to 150 cm^3^, heated to boiling, and if necessary, filtered through a medium-fast filtering paper. The precipitate (tungstic acid) was carefully washed with HC1 (1:4). A 15 cm^3^ portion of H_2_SO_4_ (1:1) was added to the filtrate and the solution was heated until fumes of SO_3_ evolved. After cooling, 100 cm^3^ of water was added, and the mixture was heated to dissolve the salts. The filtrate together with the washing was transferred into a 1000 cm^3^ volumetric flask and diluted with water to the mark.

The bottled water from three Bulgarian brands (“Devin” mineral water, “Gorna Banya” mineral water, and “Sevtopolis” table water) was bought from a local supermarket (Plovdiv, Bulgaria) and analyzed on the same day.

### 3.6. Procedure for the Determination of Molybdenum(VI)

An aliquot (1 cm^3^ for the steel analysis or 5 cm^3^ for the water analysis) of the analyzed solution was placed in a separatory funnel and the pH was adjusted to 1.4–1.5 with 0.25 mol dm^−3^ H_2_SO_4_ (the needed H_2_SO_4_ volumes were 1 cm^3^ for the steel analysis and 1.5 cm^3^ for the water analysis). Next, 1 cm^3^ of 0.1 mol dm^−3^ Na_2_EDTA solution, 1 cm^3^ of 7.5 × 10^−3^ mol dm^−3^ 4NC solution, and 0.8 cm^3^ of 2.4 × 10^−3^ mol dm^−3^ BZC solution were added, and the volume was brought to 10 cm^3^ with water. Then, 5 cm^3^ of chloroform was buretted and the mixture was shaken for 3 min. After phase separation, a portion of the organic extract was poured into the cell and the absorbance was measured at 439 nm against a blank. The Mo(VI) concentration was calculated from a calibration plot prepared by the same procedure using standard solutions.

## 4. Theoretical Section

The ground-state equilibrium geometries of the three possible structures of the anionic part of the ion-association complex were optimized at the B3LYP theoretical level using 3–21G basis functions. The charge and the spin multiplicity were set to −2 and singlet. Subsequent frequency calculations were carried out to check for the imaginary frequencies of the structures. The vertical excitation energies were calculated to make a comparison between experimental and theoretical spectra. The calculations were performed with the GAUSSIAN 03 commercial software [49]. The ChemCraft 1.8 program [50] was used for the visualization of the structures. 

## 5. Conclusions

A new liquid—liquid extraction—chromogenic system for Mo(VI) involving 4-nitrocatechol and benzalkonium chloride was studied. Optimal conditions were found for the formation and extraction of a ternary ion-association complex, (BZ^+^)_2_[Mo^VI^(4NC)O_2_(OH)_2_]. The structure of its anionic part was clarified with the help of theoretical TD DFT calculations. The complex is intensely colored and allows the determination of trace Mo(VI) in a simple and economical way, without the use of sophisticated instruments. The developed analytical procedure is characterized by a low LOD, good linearity, and high molar absorption coefficient. It is fast, selective, and robust. Its reliability is governed by the high masking efficiency of Na_2_EDTA and the wide optimal intervals of the investigated parameters.

## Figures and Tables

**Table 1:** Extraction—spectrophotometric optimization of the Mo(VI)–4NC–BZC–water–chloroform system.

Parameter	Optimization Range	Optimal Value	Figure
Wavelength, nm	UV/Vis	439	Figure 1
Concentration of 4NC, mol dm^−3^	(0.15–12.5) × 10^–4^	7.5 × 10^−4^	Figure 2
Concentration of BZC, mol dm^−3^	(0.12–2.88) × 10^−4^	1.9 × 10^−4^	Figure 2
Concentration of H_2_SO_4_, mol dm^−3^	(0.25–18.75) × 10^−2^	3.75 × 10^−2^	Figure 3
Extraction time, s	15–300	180	Figure 4

**Table 2:** Extraction characteristics.

Characteristic	Value
Extraction constant (log*K*)	9.1 ± 0.4 ^a^ 9.07 ± 0.08 ^b^ 9.11 ± 0.08 ^c^
Distribution ratio (log*D*)	1.22 ± 0.03 (*n* = 4)
Fraction extracted (*E/*%)	94.3 ± 0.4 (*n* = 4)

^a^ Mobile equilibrium method. ^b^ Holme—Langmyhr method. ^c^ Harvey—Manning method.

**Table 3:** Effect of foreign ions on the determination of 7.1 μg Mo(VI).

Foreign Ion (FI) Added	Added Salt Formula	Amount of FI Added/mg	FI: Mo Mass Ratio	Amount of Mo Found/μg	E%
Al(III)	Al_2_(SO_4_)_3_ 18H_2_O	3.55	500	7.2	102
Ca(II) ^a^	CaSO_4_	3.55	500	7.0	98.4
Cd(II)	CdCl_2_	1.42	200	7.0	98.0
Citrate ^a^	Na_3_C_6_H_5_O_7_	7.1	1000	7.1	100
Cl^− a^	NaCl	7.1	1000	7.1	100
Co(II)	CoSO_4_ 7H_2_O	0.142	20	7.1	99.8
Cr(III) ^b^	Cr_2_(SO_4_)_3_	1.78	250	7.1	99.8
Cr(VI)	K_2_CrO_4_	0.071	1	7.1	99.3
Cu(II)	CuSO_4_ 5H_2_O	1.42	200	6.9	97.0
EDTA	Na_2_EDTA	35.5	5300	7.1	99.3
F^−^	NaF	3.55	500	7.4	104
Fe(III) ^b^	Fe_2_(SO_4_)_3_	2.84	400	7.1	99.8
HPO_4_^2 a^	Na_2_HPO_4_ 12H_2_O	7.1	1000	6.9	96.6
K(I) ^a^	K_2_SO_4_	7.1	1000	7.1	100
Li(I) ^a^	Li_2_SO_4_ H_2_O	7.1	1000	7.1	100
Mg(II) ^a^	MgSO_4_ 7H_2_O	7.1	1000	7.2	101
Mn(II) ^b^	MnSO_4_ H_2_O	0.355	50	7.2	101
Ni(II) ^b^	NiSO_4_ 7H_2_O	1.42	200	7.4	104
NO_3_^−^	NaNO_3_	0.036 0.71	5 100	7.1 5.0	99.0 70.9
Re(VII)	NH_4_ReO_4_	3.55	500	7.0	99.3
Tartrate	K,NaC_4_H_4_O_6_	0.71	100	7.1	100
V(V) ^b^	NH_4_VO_3_	0.036	5	7.2	101.5
W(VI)	Na_2_WO_4_ 2H_2_O	0.007	1	9.0	126
Zn(II) ^a^	ZnSO_4_ 7H_2_O	3.55	500	7.0	98.5

^a^ Higher ion-to-Mo(VI) ratios were not studied. ^b^ In the presence of 1 cm^3^ 0.1 mol dm^−3^ Na_2_EDTA.

**Table 4:** Determination ^a^ of molybdenum in synthetic mixtures (SM) and referent standard steels (RSS).

Sample				Molybdenum Found ^a,b^/%
#	Description	Mo Content/%	Other Components/%	
SM1	1.5% manganese-molybdenum steel (synthetic mixture)	0.25	1.6 (Mn), 98.15 (Fe)	0.247 ± 0.004
SM2	Austempered ductile iron (ADI) with added nickel and molybdenum	0.37	1.5 (Ni), 98.13 (Fe)	0.369 ± 0.003
SM3	Low nickel-free stainless steel (synthetic mixture)	2.5	17 (Cr), 12 (Ni), 68.5 (Fe)	2.44 ± 0.06
SM4	Acid-resistant austenitic stainless steel (synthetic mixture)	2.5	20 (Cr), 34 (Ni), 3.4 (Cu), 40.1 (Fe)	2.50 ± 0.04
RSS1	Referent standard steel ^c^	0.96	17.7 (W), 4.21 (Cr), 1.58 (V), 0.35 (Mn), 4.71 (Co),0.081 (C), 0.18 (Si), and the balance Fe	0.955 ± 0.009
RSS2	Referent standard steel ^c^	0 ^d^	1.57 (W), 1.04 (V), 17.55 (Cr), 9.61 (Ni), 0.99 (Nb), 0.13 (Ta), and the balance Fe	0.95 ± 0.01

^a^ Four replicate determinations. ^b^ ±SD. ^c^ Supplied by KCM S.A.—Plovdiv, Bulgaria. ^d^ Added Mo(VI) corresponding to a mass fraction of 0.96%.

**Table 5:** Comparison with other liquid—liquid extraction—spectrophotometric methods for molybdenum determination.

Reagent(s)	Organic Solvent (OS)	Volume of OS/cm^3^	Acidity	Sample	Linear Range/ ng cm^−3^	LOD/ ng cm^−3^	λ_max_, nm	10^−4^*ε*/ dm^3^ mol^−1^ cm^−1^	Ref.
HTC	Chloroform	10	1 mol dm^−3^ H_2_SO_4_	Synthetic samples, steels, and reverberatory flue dust	270–2400	–	424	3.6	[39]
4NC + BTC	1,2-Dichloroethane	5	pH 1.8–4.0	Steels and ferromolybdenum	200–6700	–	445	2.38	[40]
KSCN + MTOAC+ PBITU	1-Pentanol	5	3.0–5.0 mol dm^−3^ HCl	Water, soil, and root nodule	20–1000	5	470	7.6	[41]
HMAINH	MIBK	10	(1.5–1.8) × 10^−2^ mol dm^−3^ HCl	Alloys	3000–16,000	–	410	0.5643	[42]
CHHB	Toluene	10	1 mol dm^−3^ H_2_SO_4_	Steel, water, reverberatory flue dust, and industrial effluent	Up to 2310	–	404	5.62	[43]
KSCN + CTAB	1,2-Dichloroethane	10	1.25 mol dm^−3^ H_2_SO_4_	Water, steel, reverberatory flue dust, soil, and soybean nodules	100–4200	2.39	460	4.01	[44]
HTPs + aniline	Chloroform	5	pH 5.3–5.8	Soil and pea	40–4300	12–15	530–535	3.5–3.7	[45]
DPs + APs	Chloroform	5	pH 4.1–5.9	Soils, plants, and water	300–22,000	9–10	516–534	4.16–5.35	[21]
HMPPB	Carbon tetrachloride	10	(2–6) × 10^−2^ mol dm^−3^ H_2_SO_4_	Synthetic mixtures, flue dust, and water	380–1400	100	420	5.085	[20]
DNC + TTC	Chloroform	10	(0.9–7.2) × 10^−1^ mol dm^−3^ H_2_SO_4_	–	670–6720	190	410	2.16	[46]
4NC + BZC	Chloroform	5	3.75 × 10^−2^ mol dm^−3^ H_2_SO_4_	Synthetic mixtures, steels, and water	18.6–3100	5.6	439	5.5	This work

Abbreviations: 4NC, 4-nitrocatechol; APs, aminophenols; BTC, tetrazolium blue chloride; BZC, benzalkonium chloride; CHHB, 6-chloro-3-hydroxy-2-(-hydroxyphenyl)-4-oxo-4*H*-1-benzopyran; CTAB, cetyltrimethylammonium bromide; DNC, 3,5-dinitrocatechol; DPs, dithiolphenols; HTPs, hydroxythiophenols; MAINH, 2-hydroxy-5-methylacetophenoneisonicotinoylhydrazone; HMPPB, 3-hydroxy-2-[3-(4-methoxyphenyl)-1-phenyl-4-pyrazolyl]-4-oxo-4*H*-1-benzopyran; HTC, 3-hydroxy-2-(2′-thienyl)-4*H*-chromen-4-one; MTOAC, methyltrioctyl ammonium chloride; PBITU, *N*-phenylbenzimidoyl thiourea; TTC, 2,3,5-triphenyl-2*H*-tetrazolium chloride.

## Data Availability

The datasets used and analyzed during the current study are available from the corresponding author on reasonable request. All data are in the form of tables and figures.

## Sample Availability

Not available.
